# Smartphone addiction and postural alterations in the cervical region in adolescents

**DOI:** 10.1590/1984-0462/2024/42/2023051

**Published:** 2023-12-22

**Authors:** Ticiana Mesquita de Oliveira Fontenele, Paula Pessoa de Brito Nunes, Francisco Valter Miranda Silva, Catarina Nívea Bezerra Menezes, Rodrigo Fragoso de Andrade, Daniela Gardano Bucharles Mont’Alverne, Maria Vieira de Lima Saintrain, Mirna Albuquerque Frota, Ana Paula Vasconcellos Abdon

**Affiliations:** 1 Universidade de Fortaleza, Fortaleza, CE, Brasil.; 2 Universidade Federal do Ceará, Fortaleza, CE, Brasil.

**Keywords:** Smartphone, Posture, Risk factor, Adolescents, Smartphone, Postura, Fator de risco, Adolescentes

## Abstract

**Objective:**

To evaluate smartphone addiction and postural alterations in the cervical region in adolescents.

**Methods:**

A cross-sectional study with 281 adolescents (15 to 19 years old), attending the 1^st^ to the 3^rd^ grades of High School, carried out between September and October 2019 in the city of Fortaleza, Ceará, Brazil. Data collection took place in two stages. In the first, they answered four self-administered questionnaires: sociodemographic questionnaire, health conditions and smartphone use, Nordic Musculoskeletal Symptom Questionnaire (NMQ), Self-Report Questionnaire (SRQ-20) and the Smartphone Addiction Inventory (SPAI-BR). In the second stage, they were submitted to photogrammetry using the Postural Assessment Software (SAPO) and anthropometric assessment (weight and height). The software SPSS Statistics version 23.0 was used for data analysis.

**Results:**

Of the total number of adolescents, 63.3% (n=178) showed smartphone addiction, using it for 5.8 hours (±3.5) during the week and 8.7 (±4.0) hours on the weekend. When analyzing postural alignment in the anterior view, a significant reduction in the lateral head tilt was observed when typing on the smartphone (p=0.002) compared to the anatomical position (baseline). In the lateral view, an increase in head anteriorization was observed during smartphone use (p<0.05). There was an association between smartphone addiction and head anteriorization (p<0.05).

**Conclusions:**

The use of the smartphone in the typing position causes postural alterations in the cervical region, especially in adolescents with smartphone addiction. Therefore, health promotion measures that alert adolescents to the adverse effects caused by prolonged smartphone use are necessary.

## RESUMO

**Objetivo:**

Avaliar a dependência do *smartphone* e a alteração postural da região cervical em adolescentes.

**Métodos:**

Estudo transversal, com 281 adolescentes (15 a 19 anos), que frequentavam da 1ᵃ à 3ᵃ série do ensino médio, realizado entre setembro e outubro de 2019 na cidade de Fortaleza, Ceará, Brasil. A coleta dos dados ocorreu em duas etapas. Na primeira, eles responderam a quatro questionários autoaplicáveis: questionário sociodemográfico, condições de saúde e uso do *smartphon e*, Questionário Nórdico de Sintomas Musculoesqueléticos (NMQ), *Self-Report Questi onnaire* (SRQ-20) e o *Smartphone A ddiction Inventory* (SPAI-BR). Na segunda etapa, foram submetidos a fotogrametria pelo Software de Avaliação Postural (SAPO) e avaliação antropométrica (peso e altura). Para a análise dos dados, foi utilizado o SPSS Statistics versão 23.0.

**Resultados:**

Do total, 63,3% (n=178) dos adolescentes apresentaram dependência do *smartphone*, com o uso de 5,8 horas (±3,5) durante a semana e 8,7 (±4,0) no fim de semana. Ao analisar o alinhamento postural na visão anterior, observou-se redução significativa da inclinação lateral de cabeça ao digitar no *smartphon e* (p=0,002) comparado à posição anatômica (*baseline*). Na visão lateral foi constatado aumento da anteriorização de cabeça durante o uso do *smartphon e* (p<0,05). Houve associação da dependência do *smartphone* com a anteriorização de cabeça (p<0,05).

**Conclusões:**

O uso do *smartphone* na posição de digitação causa alteração postural na região cervical, destacadamente naqueles que apresentam dependência do dispositivo. Dessa forma, são necessárias medidas de promoção de saúde que alertem os adolescentes sobre os efeitos adversos causados pelo uso prolongado do *smartphone*.

## INTRODUCTION

Smartphone use is routinely perceived in different age groups due to numerous tools offered by this device. However, adolescents use a variety of applications such as social media, games, movies, among others, which connect them to the internet for long periods of time.^1^ The number of smartphone users worldwide exceeded three billion in 2020, as it is considered the main means of accessing the internet. According to a global survey, 67.1% of the world’s population use the smartphone as the main means of accessing the internet.^2^

Studies carried out in different countries on the European and Asian continents report high smartphone use by adolescents.^3,4^ Other surveys aimed at this population warn that excessive smartphone use is a risk factor for disorders such as smartphone addiction, common mental disorders such as anxiety and depression, poor sleep quality, lower school performance and social isolation.^3,5^

From this viewpoint, smartphone addiction is characterized by the inability to control its use, which generates pleasure and reduces the pain and stress sensations. However, excessive smartphone use has negative effects on financial, physical, psychological, and social aspects of life in addition to causing musculoskeletal disorders.^6^

Smartphone addiction associated with an inharmonious body posture has negative effects on the physical health of adolescents, resulting in postural alterations, musculoskeletal discomfort, in addition to aggravating the already established alterations.^7^ The posture caused by excessive flexion when using the smartphone for prolonged periods of time is known as ‘text neck’; this term is related to the forward and down movement of the neck, with protrusion and internal rotation of the shoulders, resulting in injuries to the osteoarticular structure of the spinal column, neck, upper limbs and, consequently, pain in the region and headache.^8,9^

Even considering the aforementioned evidence, there are controversies in the studies that can elucidate the effects of smartphone use on the onset or worsening of musculoskeletal and postural alterations in adolescents. The present study aims to encourage and support the monitoring and implementation of educational measures for the conscious use of this device based on scientific evidence, which is in line with previous recommendations by the World Health Organization regarding the problematic use of screens, including the smartphone.^10^ Therefore, the aim of this study was to evaluate smartphone addiction and postural alterations in the cervical region in adolescents.

## METHOD

This is a cross-sectional study, derived from a research project entitled “Study of postural and pain alterations in the cervical region associated with smartphone use in adolescents”. Study recruitment and data collection were carried out in August and September of 2019, and the study was developed in the city of Fortaleza, Ceará, northeastern Brazil.

The data were collected at State Schools of Vocational Education (*Escolas Estaduais de Educação Profissional* — EEEPs) in the city of Fortaleza, Ceará, Brazil. The EEEPs were implemented in 2008 in Ceará. There are 115 EEEPs in the entire state, operating on a full-time basis, integrating high school with vocational education, providing a fairer future with more opportunities for young people from Ceará. In 2019, there were 21 EEEPs in the city of Fortaleza, distributed among the regional executive secretariats.^11^

In this study, the selection of adolescents was carried out in two stages. In the first stage, six schools were selected by drawing lots, one in each regional sector, with the exception of the downtown region, which does not have EEEPs. In the second stage, one class per grade (1^st^ to 3^rd^) and school shift (morning or afternoon) were chosen by drawing lots, totaling three classes per EEEPs. At least 13 adolescents were recruited per class.

A total of 281 adolescents (aged 15 to 19 years), attending the 1^st^ to the 3^rd^ grades of High School, enrolled in the 2019 school year in the selected EEEPs and who had a smartphone participated in the study, comprising a probabilistic sample with equitable distribution between the classes: 96 from the 1^st^ year, 95 from the 2^nd^ year and 95 from the 3^rd^ year of High School. As a sample loss, ten participants were removed, five due to not filling out one of the collection instruments or anthropometric data, and five due to the impossibility of analyzing posture using the SAPO software.

The exclusion criteria comprised the students who did not attend classes on the days of data collection, those with a self-reported diagnosis of scoliosis, fractures or degenerative lesions in the cervical spine, recent traumatic injuries and people with physical disabilities, as they are factors related to the presence of postural and pain alterations in the spinal column. Moreover, pregnant women, due to the physiological alterations of pregnancy, and people with visual, hearing and cognitive impairment were excluded due to the lack of adaptability of the collection instruments for this population and their self-completion.

After authorization was obtained from the school administration, recruitment of the participants started with a public call for an explanatory lecture addressed to the parents/guardians and adolescents. Afterwards, authorization was requested from the adolescents’ parents/guardians through the signature of the free and informed consent form (TCLE) and after obtaining the adolescents’ consent through the assent term, as a condition for participating in the study.

The study was approved by the Ethics Committee for Research on Human Beings of the University of Fortaleza — COÉTICA/UNIFOR, under Opinion n. 3,341,394, in line with Resolution 466/12 of the National Health Council.

After the consent, the participants underwent data collection in two stages. In the first stage, they answered four self-administered data collection instruments, in their own classrooms at school, at a time determined by the principal aiming to guarantee non-interference with the school activities.

The applied collection instruments were:

1. A sociodemographic questionnaire on health conditions and smartphone use,
2. The Nordic Musculoskeletal Symptom Questionnaire (NMQ),
3. The Self-Report Questionnaire (SRQ-20), screening for common mental disorders, and
4. The Smartphone Addiction Inventory (SPAI-BR).

The questionnaire developed by the researchers collected sociodemographic data (age, gender, race/ethnicity, social class), health conditions (upper limb dominance and use of eyeglasses/contact lenses) and data on smartphone use (time of use, position adopted during device use and device weight).

The Nordic Musculoskeletal Symptom Questionnaire is an instrument that was validated for adolescents^12^ and cross-culturally adapted to Brazilian Portuguese.^13^ It measures the report of musculoskeletal pain/symptoms (yes/no) in nine anatomical regions (cervical region, shoulders, thoracic region, elbows, wrists/hands, low back region, hips/thighs, knees and ankles/feet) in the last seven days and in the last 12 months. In this study, only pain in the cervical region was analyzed.

The SRQ-20, validated for Brazilian Portuguese, is an instrument that identifies psychosomatic symptoms for the screening of Common Mental Disorders (CMD) and is recognized by the World Health Organization and validated in Brazil. It comprises 20 questions divided into four groups of symptoms reported in the last 30 days with yes/no answers.^14^ In this study, only frequent headache complaints in the last 30 days were analyzed.

The SPAI-BR is an instrument with 26 items, divided into four subscales, with a yes/no answer to assess smartphone addiction. The cutoff point adopted for addiction was seven points, which has a sensitivity of 90.54% and a specificity of 59.93% for the validation and adaptation into Brazilian Portuguese.^15^

In the second stage, the anthropometric (weight and height) and postural (photogrammetry) assessments were carried out in a suitably adapted room at school. The body mass index (BMI) was calculated in kg/cm^2^. Weight was measured using a portable digital scale (Omron, Brazil), with a capacity of up to 150 kg, calibrated and placed on a firm surface. To measure height, an adequately calibrated portable compact stadiometer (Macrosul, Brazil) was used. BMI was classified according to the presence or absence of overweight, specific for adolescents, according to the table proposed by the World Health Organization.^16^

The postural assessment was performed by photogrammetry using the SAPO Postural Assessment Software to measure three angles, two of them in the anterior view:

1. Horizontal head alignment (HHA), which identifies the head tilt to the right (when the value is positive) or to the left (when the value is negative); and
2. Horizontal acromion alignment (HAA), which identifies the elevation of the right shoulder (when the value is negative) and left (when the value is positive);

And one in the lateral view:

1. Vertical head alignment (VHA), which identifies the anteriorization of the head and the greater the angle value, the grater the anteriorization.^17^

The performance of bilateral photogrammetry was necessary due to possible angular alterations due to upper limb dominance.^18^ For this purpose, nine markers (20 mm in diameter) were placed on the following anatomical points: glabella, manubrium, right and left tragus, chin, right and left acromion, and C7 and T1 spinous processes. The photographs were taken using a digital camera (Canon Powershot SX530HS) positioned on a tripod (Canon Nikon Sony) at a distance of three meters from the participant and a height of about half the participant’s height. The participants were instructed to remain standing up in the anatomical position and keep their eyes open, to have the photographic record performed both in the anatomical position (baseline line) and while typing on the smartphone in the usual way each participant did it.^19^

In this research, two outcomes were considered:

1. Postural alignment, measured from three angles, and evaluated in two positions (typing on the smartphone and anatomical — baseline), aiming to analyze the postural angles adopted during smartphone use; and
2. Postural alterations caused by the smartphone use, represented by the difference between the typing and the anatomical positions, represented by the delta (Δ=typing position — anatomical), which sought to analyze the influence of variables of interest on postural alterations during smartphone use.

The paired *t* test was applied to the first outcome. As for the second outcome, Student’s *t* test and Pearson’s correlation test were applied to assess the differences between postural alterations and sociodemographic variables, health conditions, anthropometric data, smartphone use and addiction. The parametric tests were selected based on the result of the Kolmogorov-Smirnov (KS) normality test. Data were analyzed using descriptive and inferential statistics using the SPSS Statistics IBM® software, version 23.0, with a significance level set at 5% (p<0.05).

## RESULTS

Regarding the sociodemographic profile, there was a higher proportion of male adolescents, or 53.0% (n=149); 31.7% (n=89) were aged 16 years old; 83.6% (n=235) were of social class D/E; and 53.4% (n=150) self-reported being of brown color. Regarding health conditions, 85.8% (n=241) were right-handed, 41.6% (n=117) wore eyeglasses/contact lenses. Of the total, 48.0% (n=135) and 53.7% (n=151) reported complaints of pain in the cervical region in the last seven days and 12 months, respectively, in relation to the data collection day, and 43.1% (n= 121) reported headaches in the last 30 days. It was found that the mean height of the adolescents was 1.65 meter (±0.09), with a mean weight of 61.8 kg (±13.4) and a body mass index (BMI) of 22.3 (±4.1). It was verified that 63.3% (n=178) of the adolescents had smartphone addiction, with a daily use of 5.8 hours (±3.5) during the week and 8.7 (±4.0) on the weekend. Regarding the position during smartphone use and the device weight, it was found that 54.1% (n=152) used the smartphone while lying down and the device weighed, on average, 156.3 (±24.1) grams (Table 1).

**Table 1:** Sociodemographic variables, health conditions and smartphone use by adolescents in full-time schools. Fortaleza (CE), Brazil, 2019.

Variables		n	%	Mean±SD
Sociodemographic				
Sex				
	Male	149	53.0	
	Female	132	47.0	
Age (years)				
	15	78	27.8	
	16	89	31.7	
	17	87	31.0	
	18	21	7.5	
	19	6	2.1	
Self-reported ethnicity				
	Brown	150	53.4	
	White	72	25.6	
	Black	39	13.9	
	Yellow	11	3.9	
	Indigenous	9	3.2	
Social class				
	A/B	9	3.2	
	C	37	13.2	
	D/E	235	83.6	
Health conditions				
Upper limb dominance				
	Right	241	85.8	
	Left	23	8.2	
	Both	17	6.0	
Wears eyeglasses/contact lenses (yes)		117	41.6	
Cervical pain complaints (last 7 days) (yes)		135	48.0	
Cervical pain complaints (last 12 months) (yes)		151	53.7	
Frequent headaches (yes)		121	43.1	
Participant’s height (meters)				1.65±0.09
Participant’s weight (kg)				61.8±3.4
Body Mass Index (BMI) (kg/m²)				22.3±4.1
Smartphone use				
Time of use (hours)				
	In the week			5.8±3.5
	On the weekend			8.7±4.0
Body position during smartphone use				
	Lying down	152	54.1	
	Sitting down	109	38.8	
	Standing	6	2.1	
	Lying down and sitting down	13	4.6	
	All three	1	0.4	
Smartphone weight (milligrams)				156.3±24.1
Smartphone addiction (yes)		178	63.3	

SD: standard deviation.

When analyzing the first outcome related to postural alignment during smartphone use, a significant reduction in lateral head inclination (HHA) was observed when typing on the smartphone (p=0.002) compared to the anatomical position (baseline) and postural alignment in the anterior view. In the lateral view, an increase in head anteriorization was observed through the VHA on the right (p=0.000) and left (p=0.000) sides when typing on the smartphone, when compared to the anatomical position (Table 2).

**Table 2:** Analysis of postural alterations related to smartphone use by adolescents from full-time schools. Fortaleza (CE), Brazil, 2019.

Alignment (degrees)		Position		p-value
		Anatomical (baseline)	Typing in the smartphone	
		mean±SD	mean±SD	
Anterior view				
	Horizontal head alignment (HHA)	1.1±2.9	0.6±3.9	0.002*
	Horizontal acromion alignment (HAA)	0.4±2.8	0.3±2.5	0.382
Lateral view				
	Vertical head alignment (VHA)			
	Right	15.5±9.3	37.4±14.3	0.000*
	Left	17.5±9.8	41.6±13.8	0.000*

SD: standard deviation. Anterior view: HHA: positive value represents head tilt to the right; HAA: positive value represents elevation of the acromion to the left. Lateral view: VHA: a higher value means higher head anteriorization.

* p<0.05.

Regarding the second outcome, no association was observed between the variables and the analyzed postural alterations (p>0.05) in the anterior view. In the lateral view, an increase in head anteriorization related to the male sex was verified by ΔVHA on the right (p=0.035) and on the left (p=0.000) sides, smartphone addiction by ΔVHA on the right (p=0.003) and on the left (p=0.002) sides, higher adolescent height according to ΔVHA on the left (p=0.000) and higher adolescent BMI according to ΔVHA on the left side (p=0.044) (Tables 3 and 4).

**Table 3:** Analysis of the relationship between postural alterations due to smartphone use and sociodemographic variables and health conditions of adolescents attending full-time schools. Fortaleza (CE), Brazil, 2019.

Variables		Postural alterations due to smartphone use (mean±SD)							
		Anterior view				Lateral view			
		ΔHHA	p-value	ΔHAA	p-value	ΔVHA right	p-value	ΔVHA left	p-value
Sex			0.230		0.328		0.035*		<0.001*
	Male	-0.3±3.6		0.0±1.7		23.6±15.8		26.9±11.6	
	Female	-0.8±2.6		-0.2±2.2		19.9±12.3		20.7±11.5	
Upper limb dominance			0.051		0.991		0.944		0.818
	Right	-0.7±3.1		-0.1±2.0		21.8±14.2		23.8±12.1	
	Left	0.6±2.4		-0.0±1.6		22.6±16.6		25.4±10.9	
	Both	0.3±4.3		-0.1±1.4		21.1±14.2		24.5±11.8	
Wears eyeglasses/contact lenses			0.419		0.388		0.441		0.777
	No	-0.4±0.3		-0.0±2.2		22.4±15.1		24.2±12.0	
	Yes	-0.7±2.7		-0.2±1.6		21.1±13.3		23.8±11.9	
Smartphone addiction			0.150		0.120		0.003*		0.002*
	No	-0.2±2.6		-0.3±2.2		18.5±14.2		21.1±11.5	
	Yes	-0.8±3.4		0.0±1.8		23.8±14.2		25.7±11.9	
Cervical pain complaints (last 7 days)			0.249		0.863		0.832		0.325
	No	-0.8±3.4		-0.0±1.8		21.7±16.1		24.7±12.4	
	Yes	-0.3±2.9		-0.1±2.2		22.1±12.4		23.3±11.4	
Cervical pain complaints (last 12 months)			0.720		0.517		0.107		0.401
	No	-0.6±2.9		-0.1±1.6		20.4±14.0		23.4±10.8	
	Yes	-0.5±3.3		-0.0±2.2		23.1±14.6		24.6±12.8	
Frequent headaches			0.983		0.676		0.625		0.602
	No	-0.5±3.4		-0.0±1.7		22.2±14.9		23.7±12.5	
	Yes	0.5±2.8		-0.1±2.2		21.4±13.7		24.4±11.2	

S.D.: standard deviation; R: right; L: left.

Δ represents the difference between the position typing on the smartphone and the anatomical position (baseline) for each variable analyzed in the table. Anterior view: horizontal head alignment (HHA) and horizontal acromion alignment (HAA). Lateral view: vertical head alignment (VHA): the higher the value, the higher the anteriorization of the head.

* p<0.05 at the *t* test.

**Table 4:** Analysis of the correlation between postural alterations due to smartphone use and the variables of interest in adolescents from full-time schools. Fortaleza (CE), Brazil, 2019.

Variables	Postural alterations due to smartphone use							
	Anterior view				Lateral view			
	ΔHHA		ΔHAA		ΔVHA R		ΔVHA L	
	r	p-value	r	p-value	r	p-value	r	p-value
Time of smartphone use during the week (hours)	0.02	0.695	0.03	0.545	-0.10	0.071	0.05	0.395
Time of smartphone use during the weekend (hours)	0.02	0.650	0.09	0.128	-0.12	0.041*	0.04	0.433
Smartphone weight (milligrams)	-0.04	0.442	-0.00	0.894	-0.03	0.574	-0.10	0.090
Height (meters)	0.06	0.272	0.10	0.070	0.08	0.168	0.20	0.000*
Body mass index (BMI)	0.03	0.619	0.05	0.381	0.12	0.044*	0.09	0.133

R: right; L: left.

Δ represents the differences between the position typing on the smartphone and the anatomical position (baseline) for each variable analyzed in the table. Anterior view: horizontal head alignment (HHA) and horizontal acromion alignment (HAA). Lateral view: vertical head alignment (VHA).

* r: Pearson’s correlation and p<0.05.

## DISCUSSION

The present research aimed to evaluate smartphone addiction and postural alterations of the cervical region in adolescents, as the addressed issue constitutes an emerging topic related to the epidemiological aspect. Among the main findings of this study, there was an increase in head anteriorization when using this device, in addition to the influence of other factors on this postural alteration, such as male gender, greater height, smartphone dependence and higher BMI. It is a condition with a negative impact on the quality of life of these young individuals and can cause early cervical pain and static postural alteration.^5^

Regarding the sociodemographic variables, the sample showed a higher proportion of male adolescents, aged 16 years, of self-reported brown color and social class D/E. Similar data were reported by Cha and Seo^1^ and Firat et al.,^4^ characterizing a similar sociodemographic profile of smartphone users, which may contribute to the generalization of the results of this study to other adolescents.

Regarding smartphone addiction, a prevalence of 63.3% (n=178) was found in the sample, with a time of smartphone use of 5.8 hours (±3.5) during the week and 8.7 hours (±4.0) on the weekend. Equivalent results were found in other studies developed in different countries, reporting high smartphone use and its association with addiction, due to the direct relationship between the two variables.^3,4^

The technologies contained in the smartphone have increasingly attracted the population to use this device. Therefore, adolescents tend to use it for several hours due to factors and demands inherent to them, such as study, socialization, leisure and work. However, prolonged use can compromise the individuals’ health.^5^

The literature reports that smartphone addiction is a behavioral disorder characterized by impulsiveness and uncontrollable use, such as frequent viewing of games, applications and notifications, resembling other behavioral addictions. Although there is no specific classification in the Diagnostic and Statistical Manual of Mental Disorders (DSM-5), this behavior has negative consequences for the adolescent biopsychosocial aspects.^20^ Moreover, as a consequence of the use of these technologies, a term linked to the modern era epidemic, called “Text Neck”, which refers to the use of the smartphone while keeping the neck in a flexed position, has emerged.^21^

From this perspective, regarding postural alignment, it was observed that the sample of adolescents showed, in the anterior view, a significant reduction in the lateral head tilt when typing on their smartphones, and in the lateral view, they adopted a cervical spine anteriorization posture (flexed posture) when typing, compared to the anatomical position (baseline). Additionally, an association was verified between alterations in the postural alignments in the lateral view caused by smartphone use and some investigated variables, whereas the anteriorization of the head was related to the male gender, smartphone addiction, complaints of neck pain in the last 12 months, greater height and BMI.

Supporting these findings, a survey conducted with 100 adolescents in South Korea showed that smartphone-addicted participants used their devices in a more flexed craniocervical posture.^22^ Prolonged smartphone use can cause greater fatigue of the cervical muscles responsible for maintaining an upright cervical posture, which results in greater cervical flexion. This posture favors the overload of myo-articular structures, such as muscles, ligaments and vertebral discs, causing inflammatory and degenerative processes.^7^

The relationship between cervical anteriorization and pain complaints verified in the present study is similar to the findings of another study carried out in Hong Kong with 560 students, which demonstrated an association between a greater craniovertebral angle and musculoskeletal pain in students with longer time of smartphone use, suggesting targeted interventions aimed to reduce the time of use, seeking to minimize the risk of musculoskeletal disorders.^9^ A systematic review study supported the findings of the present research, when investigating the prevalence of complaints, symptoms and musculoskeletal pathologies associated with the use of smartphones, indicating a high prevalence of 55.8 to 89.9% of cervical and upper back pain complaints.^23^

Regarding the association of postural alterations when using the smartphone and the participants’ gender, the study showed that males had a greater head flexion angle when using the device. To reduce the biomechanical loads on the shoulder joint and minimize the development of fatigue in neighboring muscles when using both hands, they may have lowered the smartphone and this resulted in the great head flexion angle to look at the device.^24^

As for the association of postural alteration with the participants’ height and BMI, the higher the values of these variables, the greater the head anteriorization. These findings can be explained by the associated differences between height and body weight of each individual.^25^ However, regarding the association of postural alterations with the cell phone weight, no data were found in the literature.

Despite the evidence of the association between smartphone use and postural alterations, it is noteworthy that the issue of ideal posture remains controversial among health professionals and the general public. The dichotomous relationship of ‘good’ and ‘bad’ posture, present in society’s beliefs and medical science to these days, has not been supported by the latest evidence. About this subject, the literature shows that the context (environmental and emotional factors) and individual preference of each person are determining factors to identify an appropriate posture.^26^ Complementing these findings, Bull et al.^27^ reinforce that moving and changing positions to reduce time in sustained postures are probably more important than the posture itself.

Considering the findings of the present study, the consequences of smartphone use on the health of adolescents are a matter of concern, as these individuals are still in the developmental phase. Previous recommendations warned about the use of mobile devices such as smartphones, recommending frequent breaks.^28^ The Brazilian Society of Pediatrics (*Sociedade Brasileira de Pediatria*) advises that adolescents should not spend more than two to three hours/day interacting with screen devices.^29^

It was verified that, when using the smartphone, adolescents exhibit a postural alteration in the cervical region. The angles that showed alterations had a statistically significant association with gender, participants’ height and smartphone addiction. Considering these findings, it is evident that smartphone addiction brings problems to the adolescents’ physical health, such as postural alterations in the cervical region. Based on the results found herein, educational campaigns are recommended in different socio-educational settings and in the media aiming to sensibilize and alert adolescents, parents, educators and health professionals about the risks of smartphone addiction, as well as postural changes and musculoskeletal disorders linked to overuse. Furthermore, the school, as a favorable environment for monitoring risk and protection factors for adolescents, can play an important role in raising awareness about dependence on this device and the Internet.

Among the limitations of this study, a lack of evaluation of some characteristics of daily smartphone use as reported in other studies can be identified (for instance, cervical postures in the sitting position with and without support, in addition to the standing position). The sample comprised a specific age group (15 to 19 years) of adolescents, so that the younger and older age groups could not be compared. However, the results shown in this study will contribute to the discussion of the topic and reinforce the need to carry out more research regarding the object of study, considering biopsychosocial aspects, self-perception and self-efficacy.
